# Immune Recovery after Cyclophosphamide Treatment in Multiple Myeloma: Implication for Maintenance Immunotherapy

**DOI:** 10.1155/2011/269519

**Published:** 2011-04-06

**Authors:** Amir Sharabi, Nechama Haran-Ghera

**Affiliations:** ^1^Department of Immunology, the Weizmann Institute of Science, Rehovot 76100, Israel; ^2^Department of Internal Medicine B, the Tel Aviv Sourasky Medical Center, Tel Aviv 64239, Israel

## Abstract

Multiple myeloma (MM) is a progressive B-lineage neoplasia characterized by clonal proliferation of malignant plasma cells. Increased numbers of regulatory T cells (Tregs) were determined in mouse models and in patients with MM, which correlated with disease burden. Thus, it became rational to target Tregs for treating MM. The effects of common chemotherapeutic drugs on Tregs are reviewed with a focus on cyclophosphamide (CYC). Studies indicated that selective depletion of Tregs may be accomplished following the administration of a low-dose CYC. We report that continuous nonfrequent administrations of CYC at low doses block the renewal of Tregs in MM-affected mice and enable the restoration of an efficient immune response against the tumor cells, thereby leading to prolonged survival and prevention of disease recurrence. Hence, distinctive time-schedule injections of low-dose CYC are beneficial for breaking immune tolerance against MM tumor cells.

## 1. Introduction

Multiple myeloma (MM) is a progressive B lineage neoplasia characterized by clonal proliferation of malignant plasma cells that localize in the bone marrow (BM) replacing the normal BM population. A reduced level of polyclonal immunoglobulins is a consistent feature of active MM reflecting the suppression of CD19^+^ lymphocytes that correlate inversely with the disease stage. The relationship between myeloma plasma cells and the BM microenvironment is critical for the maintenance of the disease. Tumor cells and stromal cells interact via adhesion molecules and cytokine networks to simultaneously promote progression of the disease leading to bone destruction, vertebral collapse, hypercalcemia, renal failure, hypogammaglobulinemia, and peripheral neuropathy. The disease is associated with both cellular and humoral immune deficiencies [1]. Recent studies have revealed that CD4^+^CD25^high^Foxp3^+^ regulatory T cells (Tregs), which are physiologically engaged in the maintenance of immunological self-tolerance, play critical roles for the control of antitumor immune responses. Increased numbers of Tregs were documented in peripheral blood, tumor mass, and draining lymph nodes from patients of a wide spectrum of cancers. A strong correlation exists between Treg levels and the progression of cancer. The increased number of Tregs was reported to reflect poor prognosis [2] and is associated with suppression of T cell proliferation, downregulation of proinflammatory cytokines, and involvement in tumor tolerance to self antigens. Thus, new anticancer strategies involving interference in Treg biology by means of functional disruption or numerical depletion are of critical importance. Treg depletion can lead to decreased tumor cell growth both directly by enabling anti cancer cytotoxic effects or indirectly by inducing cellular immune responses against cancerous cells. General strategies to reduce Treg functions include depletion of Tregs by chemotherapeutic drugs, blockade of Treg function by target known receptors, blockade of Treg trafficking, and combing depletion of Tregs with tumor vaccination.

Recently, we showed a correlation between increased ratios of functional Tregs and disease progression in a unique mouse model of MM that mimics the human disease [3, 4]. Low-dose cyclophosphamide (CYC) that selectively depletes Tregs reduced MM progression. Treatment of tumor-bearing mice with repeated administrations of low-dose CYC at longer time intervals (coinciding with the blocked renewal of Tregs) resulted in reduced tumor load and prevention or delay of disease recurrence. The break of immune tolerance against MM tumor cells by prolonged maintenance of transient Treg depletion will be reviewed.

## 2. Immune Abnormalities in MM Patients

The number and function of T cells subsets are aberrant in patients with MM [5, 6]. The CD4 : CD8 ratio is inverted and the helper T-cell type 1 to type 2 (Th1 : Th2) ratio among CD4 cells is abnormal [7]. In addition, the levels of expression of CD28 costimulatory molecules required for T cell activation are downregulated in T cells derived from MM patients [8]. The elevated levels of transforming growth factor (TGF)-*β*, in addition to the impaired accessory signals from Th cells, contribute to the presence of dysfunctional B cells [9]. Defective natural killer cells (NK) have also been noted in MM patients [10]. Circulating dendritic cells (DCs) from MM patients were shown to be dysfunctional, failing to upregulate costimulatory molecules required for DCs activation, which led to reduced phagocytic activity and antigen presentation [11]. It was reported that the impaired activity of DCs may be linked to the upregulation of Tregs [12]. Interestingly, in vivo expansion of Tregs could be induced in the presence of MM-specific antigens in association with reduced immune effector functions. Immunologically active compounds that promote tumor-cell survival are produced by myeloma cells. The latter include TGF-*β*, IL-10, IL-6, vascular endothelial angiogenic growth factor (VEGF), Fas ligand, Mucin 1, cyclooxygenase (Cox)-2, and related prostanoids and metalloproteins [13]. The increased numbers of Treg and the impairment and modulation of immune functions in myeloma patients suggest that breaking tolerance by chemoimmunotherapy involving transient Treg depletion and recruitment of compatible immune-derived cells could perhaps reduce tumor load and delay or prevent tumor development.

## 3. CD4^+^CD25^high^Foxp3^+^ Tregs

Subpopulations of T cells with suppressive capacities against antitumor activity of the immune system were first described in the early 1980s [14]. In the last decade, these subsets of cells were found to be naturally occurring or adoptively induced. They constitute 5–10% of naïve CD4^+^  T cells in the periphery and are anergic cells with suppressive capabilities. These cells are induced early during tumor development and were shown to contribute to tumor tolerance [15]. These cells were characterized as CD4 cells that express CD25 and the transcription factor Foxp3 (forkhead/winged helix transcription factor). The latter is essential for the suppressive phenotype of the cells [16, 17]. Also, Tregs constitutively express CTLA-4, and CTLA-4 deficiency in Tregs results in enhanced antitumor immunity [18]. The mechanisms underlying the suppressive effects of Tregs include inhibiting the activity of a variety of immune cells that are tumor specific such as CD4 and CD8 cells, B cells, NK cells, natural killer T cells (NKT), and DCs [19–21]. Both types of T cells, CD4 and CD8 cells, are considered key components against tumors [22, 23]. Further, NK cells destroy tumor cells that have reduced the expression of MHC class I but still express ligands for activating receptors of NK cells [24]. These cells are prone to inhibition by Tregs, which might lead to the continuation of tumor development and proliferation.

### 3.1. Tregs and Malignancy

Tregs, naturally occurring and inducible, play a key role in the regulation of antitumor immune response in patients with cancer. It was shown that within the tumor microenvironment the tumor itself induces naïve CD4 cells to convert into antigen-specific Tregs [15]. Many of the immune cells that reside in the tumor milieu are affected by Tregs, and thus Tregs help tumors to escape detection and elimination by the immune system. It was shown that depletion of Tregs in tumor-bearing mice resulted in the enhancement of antitumor immunity and the reduction of tumor growth [25–29]. In patients with ovarian cancer, the infiltration of CD3^+^ effector T cells was associated with a better prognosis [30], whereas the accumulation of Tregs was suggestive of poor prognosis [2]. These observations were also confirmed in non-small-cell lung cancer [31]. The accumulated data strongly suggested a new therapeutic strategy in the field of cancer aimed at eliminating Tregs or disrupting their activity.

### 3.2. Treg and MM

There is now sufficient evidence that CD4^+^CD25^high^Foxp3^+^ Tregs actively impede the antitumor immune response in cancer patients. In vivo peripheral expansion of natural naïve CD4^+^CD25^high^Foxp3^+^ Tregs were observed in large cohorts of 195 MM patients [32, 33]. Increased numbers of naturally occurring Tregs correlated with disease burden (coinciding with paraprotein levels) and increased morbidity. The frequency of double negative T cells (CD3^+^, CD4^−^, and CD8^−^ *αβ*TCR^+^) was reduced in patients with MM. CD4^+^CD25^high^ T cells from MM patients were shown to coexpress markers associated with Treg phenotype such as CTLA-4, GITR, and CD69. High serum levels of IL-10 and TGF-*β* were also observed. In both humans and animal models of MM, Tregs have been described as anergic cells, fully functional in early and late stage MM, exerting strong suppression after T-cell receptor stimulation [34, 35]. Contradictory findings concerning Treg levels and activity in MM patients were published by Prabhala et al. [36], namely, reduced CD4^+^Foxp3^+^ T cells as well as Treg dysfunction, indicating that Tregs were unable to suppress anti-CD3^+^ mediated T-cell proliferation. Whether these tested cells were coexpressing CD25^high^ was not determined in this study. Notably, it seems that differences in research strategies may account for the contradictory data in the area of MM malignancy.

The response of Tregs to tumors is illustrated in mice that lack Tregs and effectively reject tumors [26]. The involvement of Tregs in MM progression was also studied in a unique mouse model of MM (5T2MM) that developed spontaneously in BM of a very old mouse of the C57BL/KalwRij strain [37, 38]. The tumor designated 5T2MM can be maintained only by transfer of BM cells from sick mice to young syngeneic mice. The 5T2MM mouse model resembles the human disease in its main localization to the BM and in the development of hind limb paralysis (due to spinal cord compression) that occurs as an early manifestation of the disease. Further, as the disease progresses, signs of paraplegia and bone lesions take place. Increased accumulation of Tregs is observed in peripheral lymphoid organs, including lymph nodes, BM, and peripheral blood during disease progression [3, 4]. The suppressive functions of Tregs were retained, indicating that the cells were functional in their capacity to regulate immune activity. A correlation between increased Treg levels and disease morbidity was observed. Adoptive transfer of Tregs separated from thymuses of sick 5T2MM mice into 5T2MM-bearing mice serving as Treg recipients, increased severity of MM and enhanced tumor progression. Severe bone destruction, fractures of vertebra, and massive tumor cell infiltration into skeletal muscles were observed. The transfer of Treg-free thymocytes did not mediate the tumor-enhancing effects [3]. These results clearly indicated that Tregs can specifically support in vivo tumor progression.

## 4. Common Chemotherapeutic Drugs Affecting Treg Depletion

A number of chemotherapeutic agents that affect Tregs are used for preventing tumor protection by the immune system. Gemcitabine is a nucleoside analogue that inhibits DNA synthesis. In patients with colon cancer that were treated with gemcitabine, the depletion of Tregs occurred concomitantly with an increase in cytotoxic CD8 T cells [39]. However, when this drug was administered in pancreatic cancer, it did not deplete Tregs although it led to activation of naïve CD4 T cells [40]. Mitoxantrone is an anthracenedione that causes DNA strand breakup and unraveling. Its administration to patients with breast cancer resulted in reduced numbers of Tregs, but this effect was not correlative with the tumor response [41]. Fludarabine is a drug used for treating chronic lymphocytic leukemia (CLL). In vitro effects of this drug were the inhibition of Tregs expansion and sustaining the potency of cytotoxic CD8 T cells [42]. Patients with CLL treated with fludarabine responded with a preferential depletion of Tregs mainly as a result of apoptosis induction [43]. Lenalidomide is a derivative of thalidomide that was approved by the FDA for treating myelodysplastic syndrome and MM. In vitro assays showed that lenalidomide affected Tregs by reducing their expansion and function [44]. CYC is an alkylating agent that is commonly used in various antitumor protocols. In addition to the general cytotoxic effects of CYC, it was reported that doses may influence selectivity of this drug against Tregs. As a result, the clinical relevance of CYC in oncologic therapy has become stronger. Its use in this regard is discussed below.

## 5. CYC in Cancer Therapy

Therapeutic approaches for breaking tolerance towards tumor cells have been studied extensively. The depletion of Tregs is the most investigated strategy and CYC was found to have specific effects on T cells with tumor inhibiting properties [45]. In the liver, CYC is converted to the active metabolic aldophosphamide and phosphamide mustards which bind to DNA, thereby inhibiting DNA replication and causing cell death. CYC has a broad antitumor spectrum, a low niche of inducing chemoresistance, and a limited hematopoietic toxicity [46]. High doses of CYC have direct tumoricidal effects, cause immunosuppression by lymphoablation, and is used also in the clinic with the primary aim of damping ongoing immune responses in patients with autoimmune diseases. Administration of low-dose CYC (in the range of 20–150 mg/kg body weight) can paradoxically augment immune responses (demonstrated in many experimental animal models) leading to immunostimulation. Low-dose CYC acts on cycling Tregs and inhibits their suppressor function by enhancing apoptosis and decreasing homeostatic proliferation. The net outcome of CYC immunomodulation depends on the balance between immune suppressive Treg response and non-Treg effector responses. Expression of GITR and Foxp3 which are involved in the suppressive activity of Tregs is downregulated by CYC administration [47]. Studies have shown the presence of a large number of Foxp3^+^ Tregs in a variety of tumors and the enhancement of natural as well as vaccine-induced antitumor T cell responses by systematically or locally removing Foxp3^+^ Tregs [48]. A single low-dose CYC causing Treg depletion was shown to be curative by potentiating tumor-specific immunotherapy against established tumors [49]. High doses of CYC were less effective in rejecting tumor growth [50]. Treatment of neu-N mice with vaccine and low-dose CYC chemotherapy rejected tumor challenge due to the recruitment of latent pools of CD8^+^ cells to the antitumor immune response [51]. Mice bearing poorly immunogenic B16 melanoma did not evoke immunity; however, the depletion of CD4^+^CD25^+^Tregs resulted in CD8^+^ T-cell mediated rejection of secondary challenged B16 tumors [52]. CYC was shown to induce IFN-*α* production, thereby augmenting lymphocyte proliferation including CD44^high^ memory T-cell phenotype that might account for the increased antibody responses and the persistence of memory cells [53]. Low-dose CYC, in addition to its detrimental effects on Tregs, was shown to augment the antitumor immunogenicity of DC vaccine tested in B16 melanoma or C26 colon cancer [54]. 

Increased Tregs in the peripheral blood and tumor microenvironment were observed in many human malignancies. However, the current clinical application of the latter in cancer chemoimmunotherapy is limited. Oral administration of metronomic low-dose CYC regimen (100 mg at regular intervals) in advanced cancer patients induced a profound and selective reduction of Tregs and restoration of T-cell proliferation and innate killing activity by NK effector functions. An increased dose of CYC (200 mg taken daily) failed to deplete Tregs or to restore immune functions [55]. These studies validated CYC ability to induce transient Treg depletion in humans. The combination of chemotherapy and immunotherapy was tested in advanced pancreatic cancer patients. The vaccination was induced using allogeneic GM-CSF secreting cell lines alone or in sequence with CYC (250 mg/m^2^) before vaccination. Further, patients with metastatic pancreatic cancer treated with CYC followed by GM-CSF had CD8^+^ T cell responses to HLA class I-restricted mesothelium epitope, and the patients' survival was prolonged [56]. 

A phase I clinical trial in patients with metastatic carcinoma and a high Treg level in their peripheral blood tested the effect of a single intravenous infusion of CYC in three different doses (e.g. 250, 500, or 700 mg/m^2^) when administered concomitantly with a nonspecific immunotherapy using intratumoral Bacilli Calmette-Guerin (BCG). It was found in this trial that CYC at the doses tested did not modulate significantly Treg numbers and function, and the authors concluded that CYC may not represent an optimal therapy for eliminating Tregs [57]. Nevertheless, it has been reported (in many animal models) that only CYC at low doses (20–150 mg/kg body weight) could enhance immunostimulation due to the selective killing of Tregs [4, 46, 47, 51]. Hence, it should not come as a surprise that high doses of 500 and 700 mg/m^2^ in the reported trial failed to reduce the frequency of Tregs or to modulate their function; in fact, the best reduction of Tregs in this trial was observed in patients who were treated with the lowest dose of three tested, for instance, 250 mg/m^2^. In the metronomic regimen studies [55], it also was stressed that only the low daily oral dose of 100 mg (in contrast to the nonactive 200 mg dose) selectively depleted Tregs cells. Another clinical trial that followed after the frequency and function of Tregs in patients with advanced hepatocellular carcinoma who were treated with CYC at doses 150, 250, or 350 mg/m^2^ on day 1 and day 29 found that only the lower doses of CYC (150 and 250 mg/m^2^) could impair Treg number and function and unmask *α*-fetoprotein-specific T cell responses. In fact, a better and more prolonged suppression of Treg function was observed in patients treated with 250 mg/m^2^. In contrast, the reduction of Tregs in response to the high CYC dose (350 mg/m^2^) was not significant [58]. There is limited information about the kinetics and function of Tregs following CYC induced transient Treg depletion (including recovery to pretreatment levels) in patients with cancer. The duration of the blocked renewal of Tregs following CYC treatment might be important when considering repeated administrations of low-dose CYC at longer intervals using the window opportunity [4, 59].

### 5.1. Effects of a Single Low- or High-Dose CYC on 5T2MM Progression

High doses of CYC are effective in tumor therapy because of their direct cytotoxic effect and inhibitory activity against cycling cells, thus killing tumor cells but along with the depletion of immune cells. In contrast, low doses of the drug have been shown to selectively reduce the number and function of Tregs and induce antitumor-mediated effects. Within 24 hours after CYC administration to 5T2MM-bearing mice at the clinical phase of the disease (60–70 days after tumor cell challenge), both low- and high-dose CYC (100 and 200 mg/kg body weight, resp.) resulted in normalization of serum paraprotein level. In the BM cavity, plasma tumor cells were replaced with normal cell populations, in association with prolonged survival. However, a higher MM incidence (80–85%) was observed in those treated with the high-dose in comparison with the low-dose CYC (40–50%) [4]. Further, mice treated not with low-dose but with high-dose CYC still had residual 5T2MM cells, because adoptive transfer of BM cells from grossly appearing mice 170 days after initiation of treatment into young syngeneic recipients resulted in the development of disease in the BM of the latter 3-4 months afterwards [59].

Inhibitory effects of CYC on Tregs have been shown in previous studies [46, 47, 51]. Mechanistic pathways to explain the susceptibility of Tregs to CYC includes downregulated expression of the survival molecule Bcl-xL and of costimulatory CTLA-4 in CD4^+^CD25^high^ cells, and a significant decreased production of IL-2 by CD4 effector cells [4]. The inhibitory effects of low-dose CYC on Tregs are essential for accomplishing antimyeloma activities, because coadministration of Tregs 24 hours following the injection of low-dose CYC (when the cytotoxic effects of the drug were substantially diminished [46]), to 5T2MM-bearing mice, abrogated the beneficial effects of CYC on MM [4]. Selective depletion of Tregs by low-dose CYC resulted in upregulated numbers of IFN-*γ* producing NKT cells that are capable of controlling tumor growth in vivo [60] and are affected reciprocally by Tregs [61, 62]. The antitumor properties of NKT cells are linked to their ability to produce large amounts of IFN-*γ* upon activation of NKT cells [63–65]. Further, the expression of MHC class II and CD86 stimulatory molecules in DCs was up-regulated, which enhanced their function as antigen presenting cells [4, 66–68]. Once the concept of low-dose CYC was proven feasible for potentially enabling an effective immune response against MM cells, it was essential to find the most effective protocol of treatment by means of satisfactory and durable antimyeloma effects.

### 5.2. Optimal Time Schedules of CYC Treatments Affecting MM Progression

**Figure 1:**
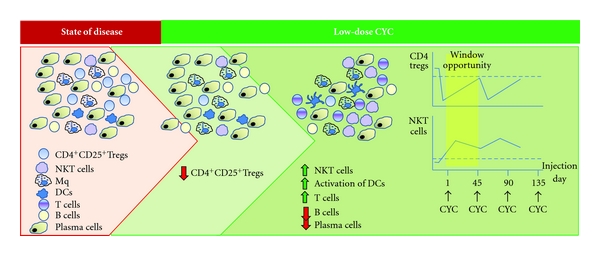
Cellular responses to low-dose CYC in mice with MM.

The initial cytotoxic effect of CYC reduced markedly tumor load, thereby eliminating the acute disease and prolonging the survival of 5T2MM-bearing mice into a chronic phase. Further, reduced MM incidence during the prolonged latency was accomplished by repeated administrations of low-dose CYC at intervals coinciding with the timing before complete Treg restoration occurred. Kinetic studies on Treg depletion and recovery to pretreatment levels following low-dose CYC injection of 5T2MM-bearing mice showed that it lasted beyond 45 after CYC administration [4]. This period of Treg recovery provided a “window of opportunity” involving a specific balance between depletion of Tregs and activation of effector T cell responses resulting in lysis of tumor cells and reduction of tumor load (Figure 1). More frequent administrations of low-dose CYC at 7- or 21-day intervals did not improve the therapeutic effect, since these mice developed MM in a higher incidence [4]. These shorter time intervals limited the “window of opportunity” by preventing recruitment of effector T cells, NKT, or DCs required for the reduction of MM incidence. Continuous low-dose CYC treatments at 45-day intervals were shown to be most effective in reducing and/or preventing MM development [4, 59]. Moreover, even when the initial injection of a high-dose CYC was chosen, further repeated injections of low-dose CYC at 45-day intervals prevented the disease [59]. Less frequent administrations of low-dose CYC enabled recruitment of compatible immune-derived cells that reduced tumor load and prolonged survival with minimal residual disease. Thus, issues of doses and schedules are crucial.

## 6. Concluding Remarks

The development of MM is associated with humoral and cellular immune deficiencies. Immunological active components that promote tumor cell survival are produced by MM cells. Increased numbers of functional Tregs correlate with MM progression and severity of disease. Thus, the effectiveness of antitumor therapy could be enhanced by the removal of Treg suppressive activity. In this regard, CYC has a broad antitumor spectrum including lymphoablation in addition to a direct tumoricidal effect. CYC-induced disruption of Tregs as a means for reducing the progression of MM depends on dosing and time schedule of drug administration. The use of high-dose of CYC is less effective in preventing the disease, since the cytotoxic effect then is less selective, reducing both tumor cells as well as immune cells with potential antitumor properties. In contrast, treatment with low-dose CYC is associated with selective Treg depletion, which leads to the restoration of peripheral effector T cell proliferation and immune function and to a reduction in MM incidence. Repeated administrations of low-dose CYC at longer intervals (coinciding with the period of blocked renewal of Tregs) enable on the one hand the elimination of tumor cells and on the other hand the breakdown of immune tolerance as a result of recovery in effector T cells, NKT cells, and mature DCs that would react against the residual tumor cells (Figure 1). The results from preclinical mouse model present immunological concepts that should be translated to a clinical setting.
